# A Novel Mode of Sympathetic Reflex Activation Mediated by the Enteric Nervous System

**DOI:** 10.1523/ENEURO.0187-20.2020

**Published:** 2020-08-05

**Authors:** T. J. Hibberd, W. P. Yew, B. N. Chen, M. Costa, S. J. Brookes, N. J. Spencer

**Affiliations:** 1 Visceral Neurophysiology Laboratory, College of Medicine and Public Health, Flinders University, 5042, Adelaide, South Australia, Australia; 2 Neurogastroenterology Laboratory, College of Medicine and Public Health, Flinders University, 5042, Adelaide, South Australia, Australia

**Keywords:** enteric nervous system, intestinofugal, motor complex, prevertebral ganglia, sympathetic nervous system, viscerofugal

## Abstract

Enteric viscerofugal neurons provide a pathway by which the enteric nervous system (ENS), otherwise confined to the gut wall, can activate sympathetic neurons in prevertebral ganglia. Firing transmitted through these pathways is currently considered fundamentally mechanosensory. The mouse colon generates a cyclical pattern of neurogenic contractile activity, called the colonic motor complex (CMC). Motor complexes involve a highly coordinated firing pattern in myenteric neurons with a frequency of ∼2 Hz. However, it remains unknown how viscerofugal neurons are activated and communicate with the sympathetic nervous system during this naturally-occurring motor pattern. Here, viscerofugal neurons were recorded extracellularly from rectal nerve trunks in isolated tube and flat-sheet preparations of mouse colon held at fixed circumferential length. In freshly dissected preparations, motor complexes were associated with bursts of viscerofugal firing at 2 Hz that aligned with 2-Hz smooth muscle voltage oscillations. This behavior persisted during muscle paralysis with nicardipine. Identical recordings were made after a 4- to 5-d organotypic culture during which extrinsic nerves degenerated, confirming that recordings were from viscerofugal neurons. Single unit analysis revealed the burst firing pattern emerging from assemblies of viscerofugal neurons differed from individual neurons, which typically made partial contributions, highlighting the importance and extent of ENS-mediated synchronization. Finally, sympathetic neuron firing was recorded from the central nerve trunks emerging from the inferior mesenteric ganglion. Increased sympathetic neuron firing accompanied all motor complexes with a 2-Hz burst pattern similar to viscerofugal neurons. These data provide evidence for a novel mechanism of sympathetic reflex activation derived from synchronized firing output generated by the ENS.

## Significance Statement

Significant interest exists in how the gut can control other body systems. Enteric viscerofugal neurons uniquely project axons out the gut wall, forming circuits with prevertebral sympathetic neurons. Long considered principally transmitting mechanosensory information, a new mechanism is demonstrated here whereby a synchronized enteric nervous system (ENS)-generated firing pattern underlying natural gut motor behavior is also relayed through populations of viscerofugal neurons. Remarkably, this caused parallel firing in sympathetic neurons in the pattern generated by the ENS. This did not require dynamic mechanical activity. The identification of this mechanism revises the current concept of sympathetic reflexes being simply distension reflexes.

## Introduction

The nature of direct communications between the enteric nervous system (ENS; located within the gut wall) and the sympathetic nervous system (that lie outside the gut wall) which control gastrointestinal motility and other homeostatic processes remains enigmatic. Anatomical and functional evidence of entirely peripheral reflex pathways involving enteric viscerofugal neurons and sympathetic prevertebral neurons abounds (Szurszewski and King, 1989; Szurszewski and Miller, 1994; Szurszewski et al., 2002; Szurszewski and Linden, 2012). These circuits are formed by enteric viscerofugal neurons whose nerve cell bodies are located in the gut wall and axons project out through extrinsic nerve trunks (Szurszewski and Miller, 1994). Viscerofugal synaptic inputs are received by postganglionic sympathetic neurons (Crowcroft et al., 1971). The axons of sympathetic neurons in turn project back into the gut wall (Costa and Furness, 1984; Messenger et al., 1994; Olsson et al., 2006).

Intracellular recordings from sympathetic neurons demonstrated that viscerofugal neurons are activated by gut distension (Crowcroft et al., 1971; Szurszewski and Weems, 1976), with preferential activation by increased gut volume (circumferential length) rather than pressure (gut wall tension; (Anthony and Kreulen, 1990; Miller and Szurszewski, 2002). Some of this volume sensitivity arises from direct mechanotransduction by viscerofugal neurons (Parkman et al., 1993; Bywater, 1994; Stebbing and Bornstein, 1994; Miller and Szurszewski, 1997) and has been described in detail (Hibberd et al., 2012b; Palmer et al., 2016). However, most activation of viscerofugal neurons is indirect via synaptic inputs from other enteric neurons (Miller and Szurszewski, 1997, 2002). Previous studies have demonstrated that viscerofugal neurons receive synaptic inputs from both ascending and descending pathways in the myenteric plexus but how these relate to motor activity is not clear.

The isolated whole mouse colon provides a unique model for studies of one particular pattern of gut motility. It displays a cyclical pattern of contractile activity that propagates along the colon and is highly dependent on activity in the ENS (Spencer et al., 2018). This pattern of activity is called the colonic motor complex (CMC), which comprises a period of organized and widespread enteric neuron activation, whereby many tens of thousands of enteric neurons are synaptically activated at the same time in a rhythmic firing pattern at ∼2 Hz. This behavior emerges from an ongoing periodic excitation of enteric neural circuits, which can be initiated by maintained distension (Barnes et al., 2014). The observation that the neural firing pattern underlying the CMC involved the participation of most myenteric neurons (Spencer et al., 2018) raises the possibility that viscerofugal neurons are also activated during this pattern.

The aims of this study were to determine the firing properties of viscerofugal neurons during naturally occurring CMCs and whether dynamic changes in gut wall circumference or muscle contraction were required for their activation. We also tested whether postganglionic sympathetic neurons are activated during the CMCs. This study suggests that assemblies of viscerofugal neurons relay with high fidelity the patterned neural activity from the ENS to the sympathetic nervous system. We present evidence that firing of multiple viscerofugal neurons are synchronized by an underlying 2-Hz discharge pattern of the ENS during CMCs, leading to a similar pattern of discharge in sympathetic neurons. Furthermore, this activation occurs independent of dynamic changes in intracolonic volume (filling), muscle contraction, or the expulsion of fluid along the colon, suggesting that it is driven by active enteric motor circuits.

## Materials and Methods

Mice of either sex (C57BL/6; 6–12 weeks old) were killed by isoflurane overdose followed by exsanguination (ethics no. 467/17). Following a midline laparotomy, the entire large intestine from caecum to terminal rectum was removed along with the pelvic plexuses and in some preparations, the inferior mesenteric ganglion. Tissue was immediately placed in a Sylgard-lined glass Petri dish filled with warmed (32–36°) Krebs solution (118 mm NaCl, 4.7 mm KCl, 1.0 mm NaH_2_PO_4_, 25 mm NaHCO_3_, 1.2 mm MgCl_2_, 11 mm D-glucose, and mm 2.5 CaCl_2_; gassed with 95% O_2_-5% CO_2_). The caecum was removed, and the colon was cleared of content by a combination of spontaneous emptying and flushing with Krebs solution. Preparations were further dissected depending on the experiment.

### Electrophysiological recordings of viscerofugal neuron axons during the motor complex

The firing behavior of viscerofugal neurons was recorded during CMCs in three types of preparation: (1) organ cultured flat-sheet preparations of colon; (2) fresh flat-sheet preparations of colon; and (3) fresh intact tubular preparations of colon. In both organ-cultured and fresh flat-sheet preparations, the full-length colon was cut along the anti-mesenteric border to create a flat-sheet. No longitudinal cuts were made in intact tube preparations. In all preparations, several (2–6) rectal nerve trunks on either side of the gut were isolated from surrounding connective tissue. Preparations were transferred to an organ bath for electrophysiological recordings. Fresh preparations were transferred immediately, while organ cultured preparations were maintained in culture media for 4 d (see “organotypic culture”, below) before transfer. Flat-sheet preparations were placed in a 20-ml organ bath, and uniformly stretched to a circumferential length of ∼5–7 mm with serosa uppermost by entomological pins. Tube preparations were placed in a 40-ml organ bath with an incompressible stainless-steel rod of 2.2 mm in diameter (∼ 6.9-mm circumference) inside the lumen. For comparison, six- to eight-week-old C57BL/6 mice had natural pellet diameters ranging 1.46–3.02 mm (mean 2.01 ± 0.21 mm; Hibberd et al., 2018b).

In all preparations, rectal nerve trunks were pinned to the base of the organ bath by small tungsten pins (25–50 μm in diameter) and isolated in paraffin oil. Organ baths were located on a heated base and maintained at a temperature of 35–36°C inside a Faraday cage. Preparations were constantly superfused with oxygenated Krebs solution at a rate of ∼ 3.5 ml/min. Action potentials were recorded from up to three rectal nerve trunks simultaneously by separate platinum electrodes. Neurogenic spike bursts were recorded from smooth muscle by an extracellular suction electrode (Hibberd et al., 2017) applied to the serosal layer 20–25 mm from the terminal rectum. These events comprise the electrical activity in smooth muscle underlying CMCs (Hibberd et al., 2017, 2018a; Spencer et al., 2018) and for simplicity are referred to here as CMCs. Silver chloride pellets located in the Krebs solution were used for reference electrodes to both nerve and muscle electrophysiological recordings. Nerve recording signals were amplified (ISO80; WPI) and digitized at 20 kHz (PowerLab 16/35, LabChart 8, ADInstruments). Muscle recording signals were amplified (DAM-50; WPI) and digitized at 1 kHz.

### Electrophysiological recordings of sympathetic efferent axons during the motor complex

**Figure 1.**
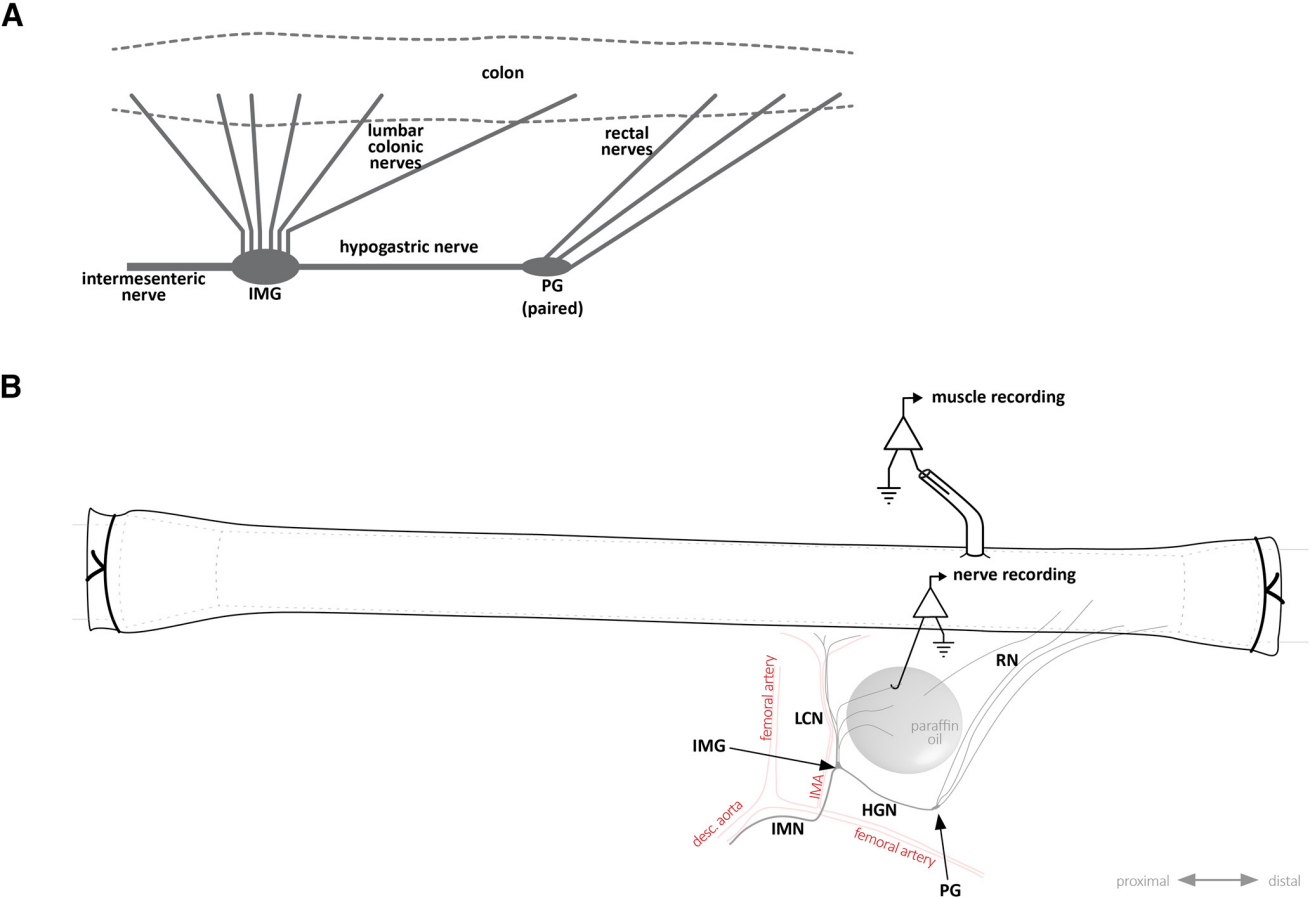
Nerve pathways between colon and the inferior mesenteric ganglion. ***A***, Simplified schematic diagram showing the nerve connections between the colon, inferior mesenteric ganglion, and pelvic ganglia. While pelvic ganglia are always paired, the inferior mesenteric ganglion was either an unpaired single ganglion, or more commonly, two separate ganglia, each of which associated with one of the hypogastric nerves. ***B***, Schematic diagram showing a colonic tube preparation with connections to the pelvic ganglia and inferior mesenteric ganglion as typically arranged in an organ bath for recording. Note the diagram shows the most common arrangement featuring a paired IMG with a single connecting hypogastric nerve trunk. For simplicity, the other IMG is not shown. The centrifugal processes of cut lumbar colonic nerves, which could be seen emerging from the IMG, were isolated in paraffin oil for sympathetic nerve recordings, as shown. Conversely, rectal nerve recordings (of viscerofugal neuron activity) were made from the peripheral sides of cut nerves. Smooth muscle electrical activity was recorded by suction electrode within ∼5 mm of recorded rectal nerve entry to the gut wall (15–20 mm from the terminal rectum). IMG, inferior mesenteric ganglion; LCN, lumbar colonic nerves; PG, pelvic ganglia; HGN, hypogastric nerve; RN, rectal nerve; IMN, intermesenteric nerve; IMA, inferior mesenteric artery.

The firing behavior of postganglionic sympathetic efferent neurons was recorded in fresh intact tube preparations that contained both the pelvic plexuses connected to the inferior mesenteric ganglion. These preparations were set up as described for tube preparation above. However, nerve recordings were made from the central cut endings of lumbar colonic nerve trunks close to the ganglion and thus reflect firing in sympathetic postganglionic axons (Fig. 1).

### Organotypic culture

Organ cultured preparations were maintained in sterile culture medium [DMEM/Ham’s F12, Sigma (1:1 ratio mix, supplemented with L-glutamine and 15 mmol l^−1^ HEPES); including 10% fetal bovine serum, 1.8 mmol l^−1^ CaCl_2_, 100 IU ml^−1^ penicillin, 100 μg ml^−1^ streptomycin D, 2.5 μg ml^−1^ amphotericin B, and 20 μg ml^−1^ gentamycin; Cytosystems] and slowly agitated for 4–5 d in a humidified incubator (36°C, 5% CO_2_ in air). To prevent strong contractions from tearing the tissue during incubation, culture medium contained 1 μm hyoscine (hydrobromide; S0929, Sigma) and 1 μm nicardipine (hydrochloride; N7510, Sigma). Culture medium was replaced every 24 h. This procedure allowed degeneration of both spinal afferent neuron axon and autonomic efferent neuron axons in the guinea pig colon, while viscerofugal neurons persisted (Hibberd et al., 2012a).

### Biotinamide tracing

A bubble of biotinamide solution (5% biotinamide (N-[2-aminoethyl] biotinamide hydrobromide), dissolved in artificial intracellular solution (150 mmol/l monopotassium L-glutamic acid, 7 mmol/l MgCl_2_, 5 mmol/l glucose, 1 mmol/l ethylene glycolbis(b-aminoethyl ether)-N,N,N=,N=-tetraacetic acid, 20 mmol/l HEPES buffer, 5 mmol/l disodium adenosine triphosphate, 0.02% saponin, 1% dimethyl sulfoxide, 100 IU/ml penicillin, 100 μg/ml streptomycin, and 20 μg/ml gentamycin sulfate) was placed on a dissected nerve trunk and normal Krebs solution in the main chamber was replaced with sterile culture medium (described above). Preparations were incubated overnight (36°C, 5% CO_2_ in air). The preparations were fixed overnight in paraformaldehyde (4% in 0.1 m phosphate buffer, pH 7.0). Preparations were cleared using 0.5% Triton X-100 in 0.1 m PBS [0.15 m NaCl, pH 7.2; 3 × 10-min washes, and then washed in PBS (3 × 10-min washes) followed by incubation for 3 h in 3–1-O-(2-cyanoethyl)-(N,Ndiisopropyl) indo-carbocyanine (CY3)-conjugated streptavidin]. Preparations were then washed with PBS (3 × 10 min) and equilibrated in a series of carbonate-buffered glycerol solutions (50%, 70%, and 100% solutions; 3 × 10 min) before mounting on glass slides in buffered glycerol (pH 8.6). Confocal images were captured using a Zeiss LSM880 confocal microscope with a 20× objective lens. Z-stacks were scanned at 1.25-μm steps through the full thickness of preparations. The z-stacks were processed to obtain maximum intensity projections using ImageJ (v1.52a; National Institutes of Health).

### Immunohistochemistry

Following biotinamide tracing, a subset of control and organ-cultured preparations were additionally assessed for immunoreactivity for calcitonin gene-related peptide (CGRP), expressed in the majority of colorectal spinal afferent neurons (Robinson et al., 2004; Christianson et al., 2006), and tyrosine hydroxylase (TH), a marker of sympathetic neurons (Jobling and Gibbins, 1999; Kaestner et al., 2019). Preparations were incubated with primary antisera for CGRP (rabbit; Peninsula; IHC 6006; RRID: AB_2314156; 1:2000 dilution) and TH (chicken; Abcam; AB76442; RRID: AB_1524535; 1:200 dilution) for 48 h at room temperature, followed by PBS washing (3 × 10 min) and 4-h incubation in secondary antisera (Alexa Fluor 488; donkey anti-rabbit; Thermo Fisher Scientific; A21206; RRID: AB_2535792; 1:1000 dilution; and Alexa Fluor 647 donkey anti-chicken; Jackson ImmunoResearch; 703-605-155; RRID: AB_2340379; 1:1000 dilution). Preparations were then washed in PBS and equilibrated in carbonate/bicarbonate-buffered glycerol solutions for mounting on glass slides, as described above. Confocal images were captured using a Zeiss LSM880 confocal microscope with a 20× objective lens using identical settings for both control and cultured preparations. Z-stacks were scanned at 1-μm steps through the full thickness of preparations. The z-stacks were processed to obtain maximum intensity projections using ImageJ.

### Experimental design and statistical analysis

Simultaneous smooth muscle and nerve recordings were allowed at least 60 min for control recordings before addition of drugs. All instances of CMCs were included for analysis of smooth muscle and nerve firing rates (analyzed using LabChart 8; ADInstruments). For analysis of nerve firing, single units were discriminated by spike amplitude, duration and interspike interval using LabChart 8 Spike Histogram software (ADInstruments). The entire duration of CMCs and 120-s pre-CMC and post-CMC were sampled to determine firing rates. To analyze single unit contributions to burst firing sequences, all detectable bursts of firing within all instances of CMCs were individually selected and included for analysis. Action potentials and electrical oscillations in smooth muscle recordings were discriminated to determine average firing rates using manually set thresholds and median filtering using LabChart 8 (ADInstruments). To analyze the relationship between sequences of burst firing in rectal nerves and CMCs, the time point of each burst firing sequence was recorded while blinded to CMC recordings. Their latency to the nearest CMC was compared with those of 100 randomly generated times within the recording period for each preparation. Statistical analysis was performed on preparation averages by ANOVA (one-way with repeated measures), or Student’s two-tailed *t* test for paired or unpaired data using Prism 8 (GraphPad Software, Inc). Degree of statistical significance was given as *p* values. However, where comparisons are made, we have provided Gardner–Altman estimation plots to focus on effect sizes and confidence intervals (Fig. 5; Gardner and Altman, 1986; Ho et al., 2019). The estimation plots employ a secondary axis showing the size differences between groups as a mean and 95% confidence interval. For paired comparisons, this is the within-group difference. All data are presented as mean ± SEM unless otherwise stated. Lower case *n* always indicates the number of animals.

### Drugs

Stock solutions of drugs were made as follows: 10^−1^ m hexamethonium chloride in water (H2138; Sigma), 10^−2^ m nicardipine hydrochloride in water (Sigma; N7510), 10^−2^ m N-Vanillylnonanamide (synthetic capsaicin) in ethanol (V9130, Sigma), and 10^−1^ m 1,1-dimethyl-4-phenylpiperazinium iodide (DMPP) in water (D5891, Sigma). Drugs were kept refrigerated and diluted to working concentrations in Krebs solution, shortly before use.

## Results

### Viscerofugal neurons recorded in fresh preparations

**Figure 2.**
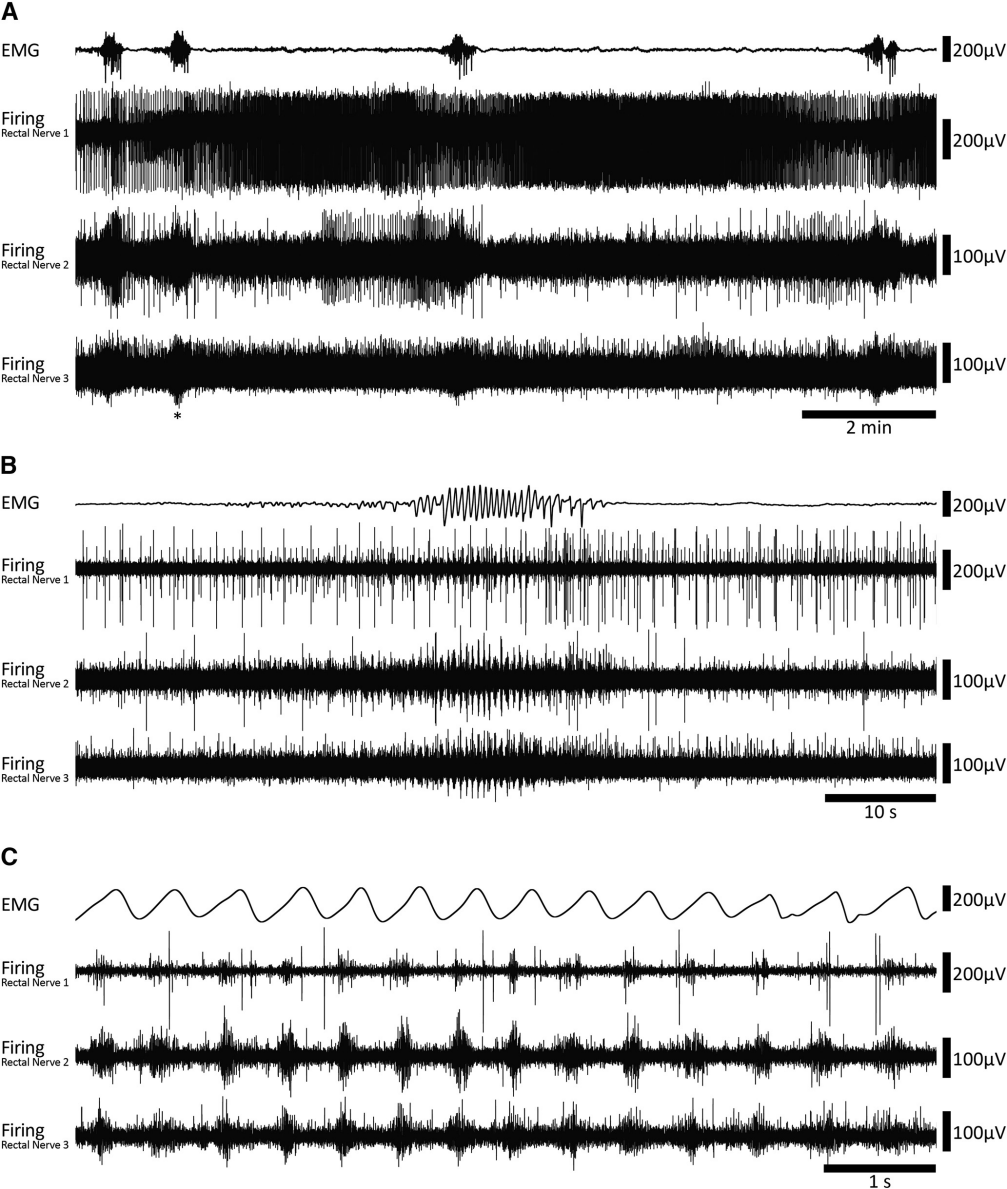
Coordinated burst firing in rectal nerves during motor complexes. ***A***, Colonic EMG and rectal nerve recording traces showing ∼15 min of ongoing motor complexes and firing activity. Four motor complexes can be seen in the EMG trace. ***B***, A single motor complex corresponding to the event marked by an asterisk below nerve 3 voltage trace in ***A***. At this timescale, oscillations in the EMG trace and bursts of firing in the nerve recording traces are evident. There are also several larger amplitude spikes in nerve recording traces 1 and 2 that do not participate in the burst firing activity. ***C***, A further expanded view of the motor complex shown in ***B***. Here, the bursts of firing are clearly seen. Furthermore, burst firing appears coordinated between each of the three rectal nerves and EMG oscillations.

Evidence suggests that viscerofugal neurons relay mechanosensory afferent information from the gut to sympathetic neurons such as gut distension (Crowcroft et al., 1971; Bywater, 1994; Stebbing and Bornstein, 1994). In this series of experiments, simultaneous rectal nerve and smooth muscle recordings were performed in eight fresh colon preparations under isometric conditions. Fresh flat-sheet (*n* = 4) and intact tube (*n* = 4) preparations behaved similarly and are therefore presented here together. Ongoing CMCs were detected in all preparations from EMG recordings (Fig. 2*A*) under control conditions. They also persisted during smooth muscle paralysis using the L-type calcium channel antagonist, nicardipine (3 μm; 6/6 preparations tested). CMCs had an average frequency of 0.35 ± 0.06 cpm (*n* = 8). Within CMCs, smooth muscle electrical activity was characterized by rhythmic voltage oscillations with or without action potentials at a mean frequency of 1.8 ± 0.1 Hz, for a duration of 33.8 ± 4.9 s (*n* = 8; Fig. 2*B*,*C*).

**Figure 3.**
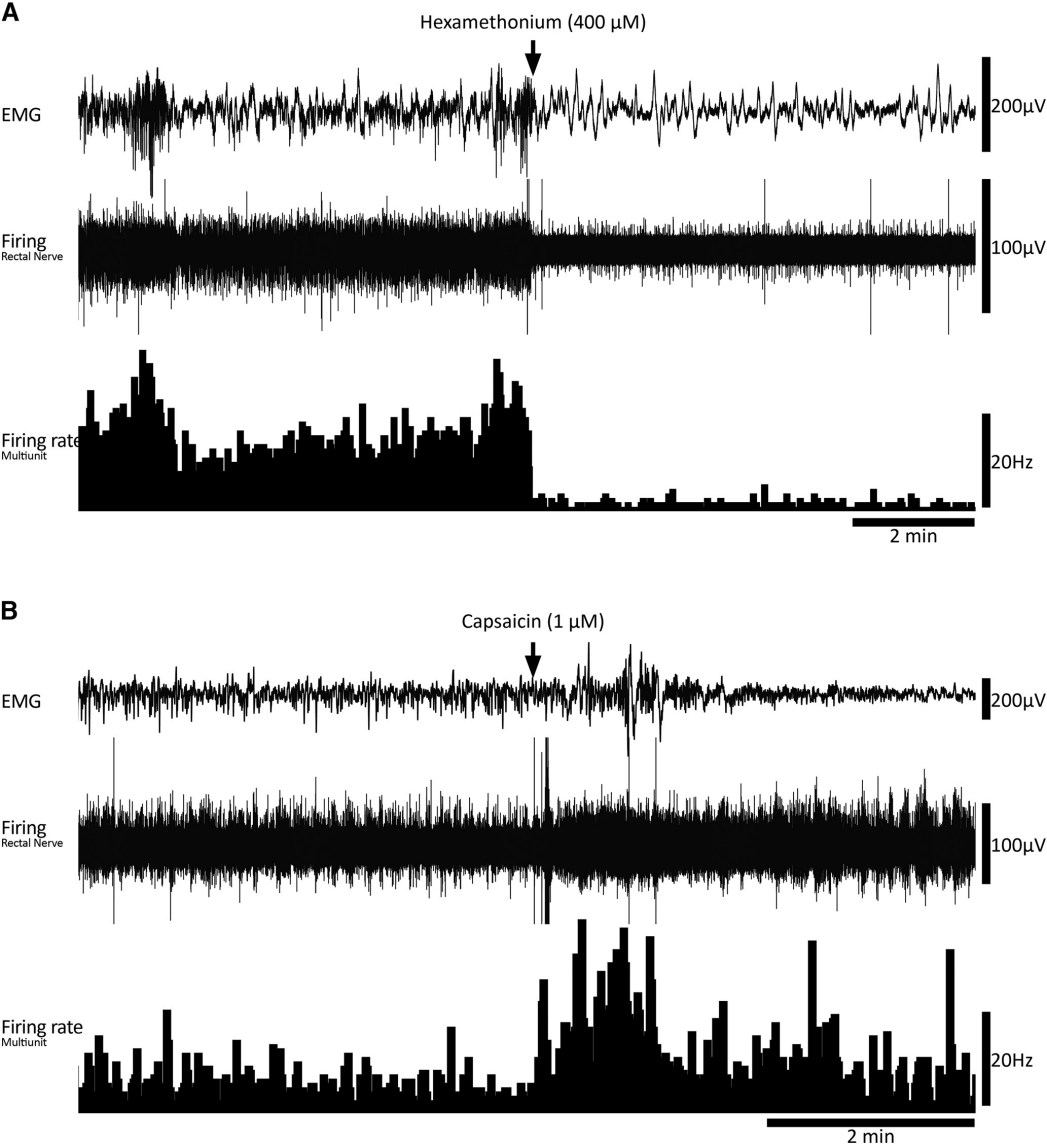
Drug responses in fresh preparations. ***A***, Example of the prompt abolition of motor complexes and large reduction in rectal nerve firing by nicotinic receptor antagonist, hexamethonium. Neither burst firing, nor motor complexes occurred in hexamethonium (*n* = 7). ***B***, Example of capsaicin-evoked firing in a fresh preparation, consistent with the presence of spinal afferent neurons.

All preparations had detectable ongoing action potential firing in rectal nerves, which was recorded during 266 individual CMCs (*n* = 8; Fig. 2*A*). Sequences of burst firing in the rectal nerves were readily observed during 134 of the 266 CMCs (54 ± 14% per preparation, on average; Fig. 2*B*). Burst firing had an average frequency of 1.9 ± 0.03 Hz (interburst interval 518 ± 18 ms) and typically aligned with muscle voltage oscillations during the CMC (Fig. 2*C*). Burst firing was significantly associated with CMCs; bursts occurred 13 ± 6 s (*n* = 8) from a CMC, compared with 122 ± 13 s for randomly generated times in the same period (*p* = 0.013, paired samples *t* test, *n* = 8). The proportions of CMCs associated with burst firing was not significantly different between control conditions (53 ± 14%) or nicardipine (3 μm; 63 ± 17%, *p* = 0.584, independent samples *t* test, *n* = 8). The persistence of burst firing in nicardipine suggests the burst firing activity accompanies CMCs and this relationship does not require muscle contractility. A bolus of capsaicin (1 μm) always evoked a barrage of action potentials in rectal nerves lasting several minutes but did not abolish CMCs or the associated burst firing in rectal nerves (*n* = 8; Fig. 3). At the end of experiments, burst firing and CMCs were always abolished by hexamethonium (400 μm bath concentration, 7/7 preparations tested; Fig. 3). In a single preparation, hexamethonium was washed out, allowing CMCs and burst firing to return. These data suggest the burst firing activity in rectal nerves is either dependent on nicotinic input, CMCs, or both.

### Viscerofugal neurons recorded after organ culture

**Figure 4.**
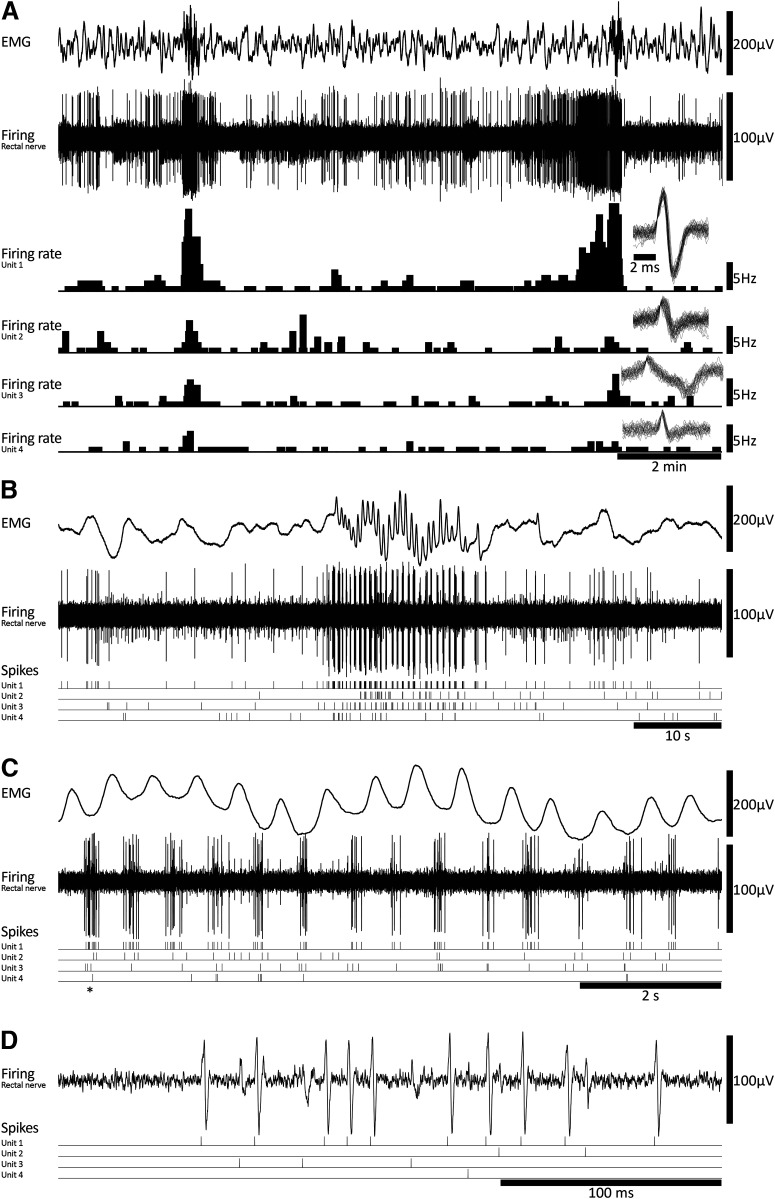
Coordinated burst firing in rectal nerves during motor complexes after organ culture. ***A***, Colonic EMG and rectal nerve recording showing ∼15 min of ongoing activity in an organ cultured preparation, including two motor complexes. The firing rates and spike shapes of four single units discriminated from the rectal nerve recording are shown. Most single units showed an increase in firing rate during motor complexes. ***B***, A single motor complex, revealing burst firing behavior in the rectal nerve. Several units contributed to the burst firing behavior; their individual spike events are indicated below the nerve recording trace. ***C***, The same event shown in ***B*** with an expanded timescale, showing in more detail the individual contributions to burst firing made by four single units. ***D***, An individual burst corresponding to the event marked by an asterisk in ***C***.

**Figure 5.**
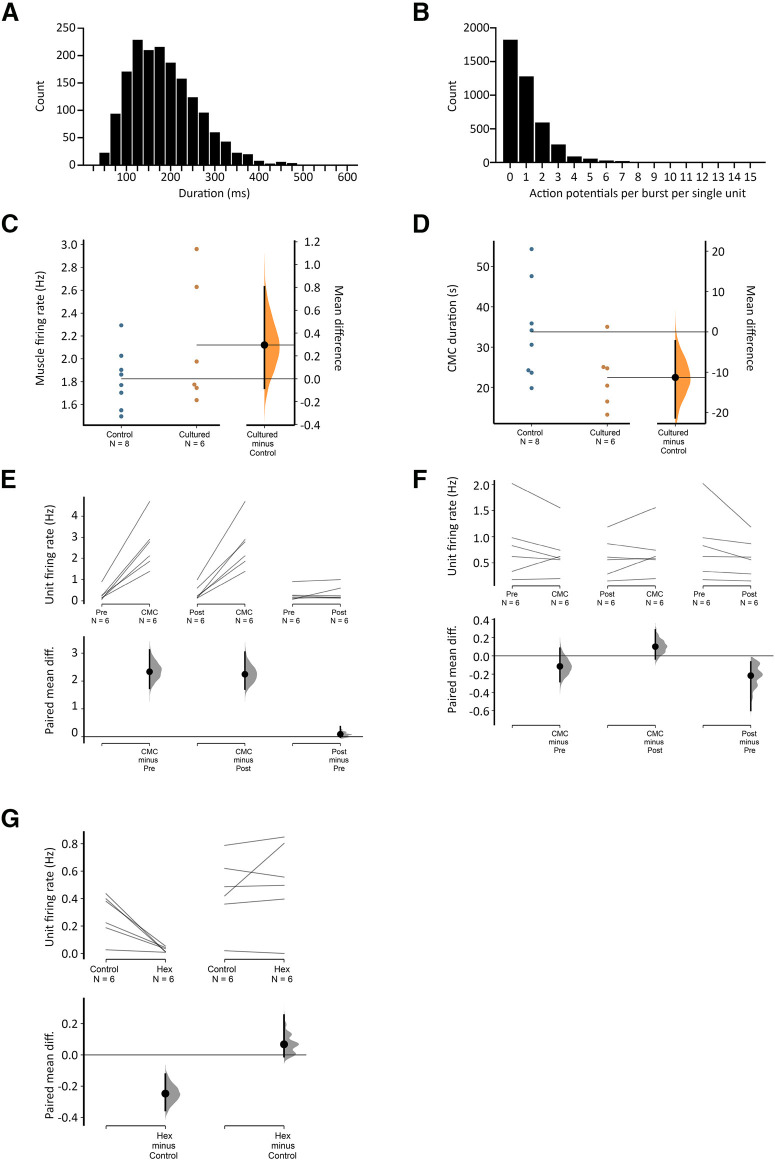
Viscerofugal neuron burst firing characteristics. ***A***, The duration of 1676 individual bursts of firing in rectal nerves during 31 motor complexes in six organ cultured preparations shown as a frequency distribution. ***B***, Distribution of the average numbers of action potentials each unit contributed to each burst in rectal nerves. Single units did not contribute to every instance of burst firing in rectal nerves, as may be seen in Figure 4. This highlights the importance of synchronization among multiple neurons, since single units alone were unlikely to encode the entire burst firing pattern. Single units contributed between 0 and 12 action potentials to a burst of firing. ***C***, ***D***, Comparisons of CMC smooth muscle firing rate and duration in control and cultured preparations, showing no significant differences between the two groups (*p* = 0.200 and 0.069, respectively, independent samples *t* test). Estimated mean differences between the groups are shown in a separate graph to the right. ***E***, ***F***, Average single unit firing rates before, during and after CMCs among units that contributed to burst firing behavior (***E***) and those that did not (***F***). All data are paired, and mean differences are shown graphically below mean firing rates. Firing in single units increased by 1–3 Hz among those that contributed to burst firing during the CMC compared with before or after CMCs, but not among those that did not contribute to burst firing (***F***). See text for details. ***G***, The effect of hexamethonium on ongoing firing rate was significant among contributing but not non-contributing burst firing units.

We sought to test whether axon firing recorded in rectal nerves originated from enteric viscerofugal neurons or extrinsic afferents whose cell bodies are located outside the gut wall (primarily in dorsal root ganglia). To test this, we cultured isolated whole mouse colons for 4 d, which causes degeneration of extrinsic axons and preserves enteric neurons and their axons (Hibberd et al., 2012a; *n* = 6). CMCs occurred in all organ cultured preparations (*n* = 6). In two preparations, CMCs occurred “spontaneously,” and in all preparations, they could be evoked by focal application of DMPP (5 μl of 10^−4^ m) onto the colorectum by hand pipette. CMCs consisted of voltage oscillations with an average frequency of 2.1 ± 0.2 Hz and a mean duration of 22.5 ± 3.4 s (*n* = 6; Fig. 4). These values were similar to CMCs in fresh preparations (*p* = 0.200 and 0.069, respectively, independent samples *t* test; Fig. 5*C*,*D*, respectively).

All preparations had ongoing action potential firing in rectal nerves (*n* = 6). Similar to fresh preparations, CMCs were accompanied by coordinated bursts of firing in rectal nerve trunks. Bursts of firing had a similar frequency as muscle voltage oscillations (2.4 ± 0.3 Hz, *n* = 6; Fig. 4). Individual bursts had an average duration of 160 ± 16 ms (1676 individual bursts, 31 CMCs, *n* = 6; Fig. 5). Single units were more readily discriminated in rectal nerve recordings of organ cultured preparations, possibly because of a decreased interference from spinal afferent action potentials. In total, 41 single units were discriminated (11 nerves, *n* = 6). Of these, 29 units showed bursts of firing accompanying CMCs. Their average firing rate was significantly increased during CMCs (2.6 ± 0.5 Hz, 33 CMCs, 29 units, *n* = 6), compared with their firing rates before and after CMCs (0.3 ± 0.1 and 0.4 ± 0.2 Hz, respectively, *p* = 0.003 and 0.003, compared with firing rates during CMCs, one-way repeated measures ANOVA, Tukey’s *post hoc* test, *n* = 6; Fig. 5*E*). Single units varied widely in their contributions to burst firing. Some single units contributed a single action potential to a minority of individual bursts. Other units contributed multiple action potentials to the majority of bursts. Overall, the average contribution from a single unit was 1.1 ± 0.2 action potentials per burst of firing (range: 0–12 action potentials per burst; 29 units, 1676 bursts, *n* = 6). The distribution of these data is shown in Figure 5. The remaining 12/41 units did not contribute to the burst firing associated with CMCs. Their firing rate during CMCs (0.7 ± 0.2 Hz) was similar to their firing rates before or after CMCs (0.8 ± 0.3 and 0.6 ± 0.2 Hz, respectively, *p* = 0.285, one-way repeated measures ANOVA, *n* = 6; Fig. 5*F*).

Burst firing never occurred in the presence of hexamethonium (400 μm; *n* = 6). However, it should be noted that hexamethonium also blocked CMCs. Hexamethonium caused a non-significant reduction in average firing rate across all units (0.4 ± 0.1 vs 0.2 ± 0.1 Hz in control and hexamethonium, respectively, *p* = 0.088, paired *t* test, 41 units, *n* = 6). The effect of hexamethonium on single unit firing rate was significant among those which contributed to burst firing (0.3 ± 0.1 vs 0.03 ± 0.01, control vs hexamethonium, respectively, *p* = 0.012, paired *t* test, 29 units, *n* = 6), but not in those which did not contribute to burst firing (0.4 ± 0.1 vs 0.5 ± 0.1 Hz, control vs hexamethonium, respectively, *p* = 0.347, paired *t* test, 12 units, *n* = 6; Fig. 5*G*). Consistent with the degeneration of functional spinal afferent axons, firing rates were not significantly affected by capsaicin (1 μm; 0.2 ± 0.1 vs 0.1 ± 0.1 Hz, control vs capsaicin, respectively, *p* = 0.193, paired *t* test, 41 units, *n* = 6), and there was no differential effect of capsaicin based on contribution to burst firing behavior. Taken together, these data are compatible with enteric viscerofugal neurons being responsible for the burst firing behavior associated with CMCs that was detected in rectal nerve trunks.

**Figure 6.**
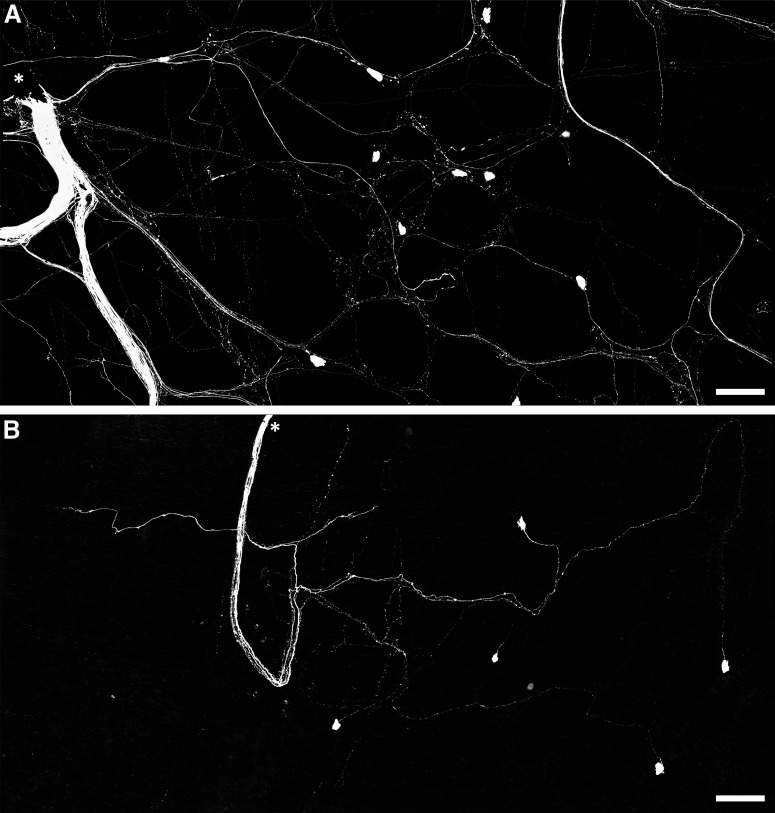
Persistence of viscerofugal nerve cell bodies in organ cultured preparations. ***A***, Confocal micrograph showing biotinamide neuronal tracing from a rectal nerve trunk in a fresh preparation. The labeled nerve trunk is indicated by an asterisk. Numerous large axons and smaller varicose fibers can be seen coursing throughout the myenteric plexus in all directions. Viscerofugal nerve cell bodies were also labeled. ***B***, Biotinamide neuronal tracing from rectal nerves after organ culture also revealed viscerofugal nerve cell bodies but a substantially reduced density of fine varicose fibers. Calibration, 100 μm.

**Figure 7.**
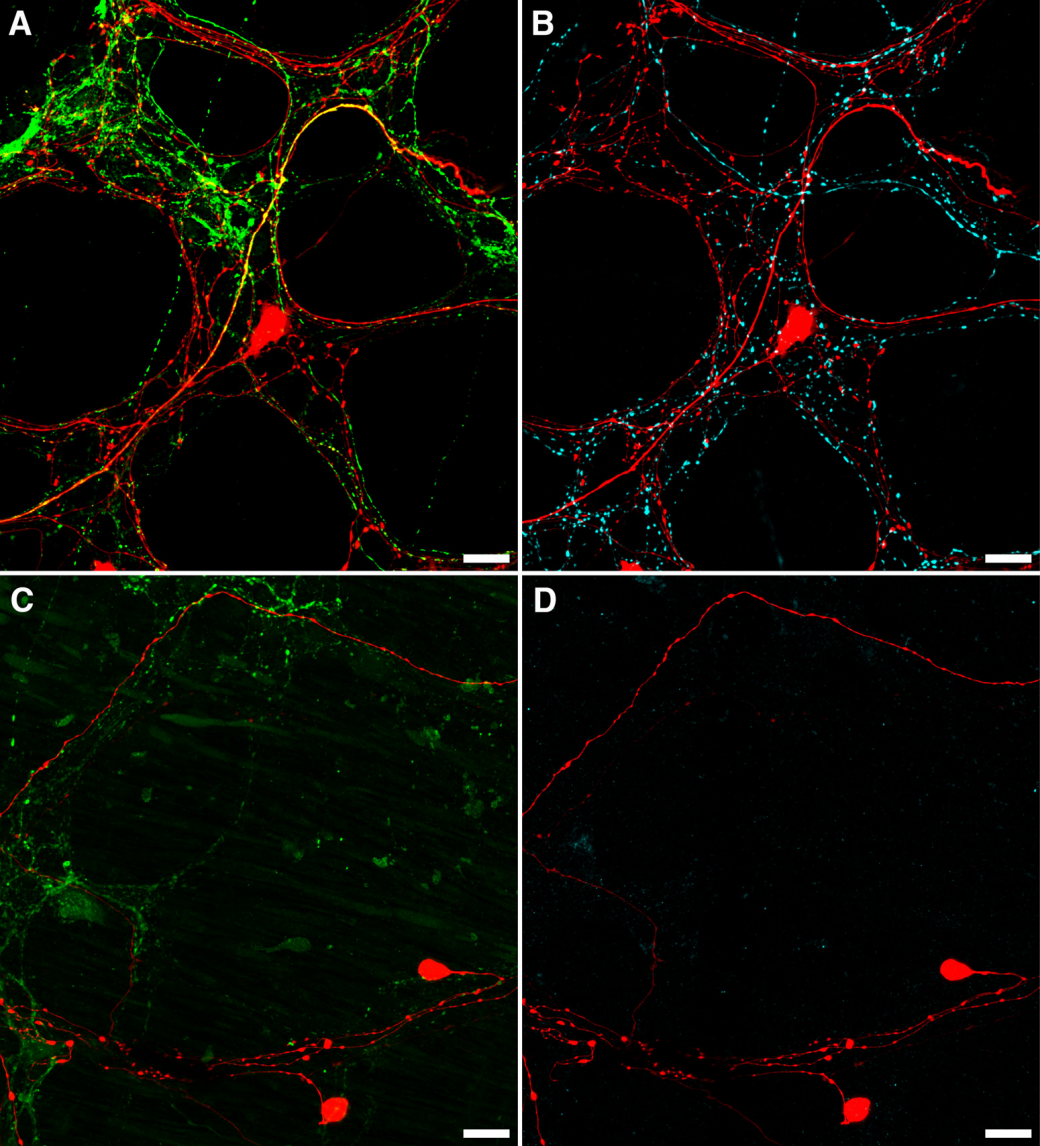
Loss of CGRP and TH after organ culture. ***A***, ***B***, An example of CGRP (***A***; green) and TH (***B***; cyan) immunofluorescence in the same biotinamide labeled (red) control preparation. Numerous varicosities containing TH or CGRP can be seen within the myenteric plexus, with some co-labeling of biotinamide-labeled axons and varicosities apparent with CGRP (yellow; ***A***), but not TH (*n* = 3). This is consistent with the presence of spinal afferent axons in control preparations. In organ cultured preparations, immunohistochemically detectable CGRP (***C***; green) and TH (***D***; cyan) were dramatically reduced, showing degeneration of extrinsic nerve fibers, while viscerofugal nerve cell bodies persisted (biotinamide; red). Additionally, no co-labeling of these markers occurred with biotinamide-labeled axons. Expectedly, faintly CGRP-immunoreactive varicosities and nerve fibers persisted in organ cultured preparations. This is consistent with a population of intrinsic enteric neurons. Together, these data support a viscerofugal origin of the activity recorded from rectal nerve trunks in organ cultured preparations.

Rapid neuroanatomical tracing with biotinamide was performed in both organ cultured (*n* = 5) and fresh preparations (*n* = 4). Biotinamide-labeled nerve cell bodies of viscerofugal neurons were identified in all preparations (Fig. 6, *n* = 9). However, cultured preparations showed a large reduction in labeled axons compared with fresh preparations, particularly fine varicose fibers characteristic of spinal afferent and sympathetic efferent neurons. Further confirmation was provided by immunohistochemical labeling of CGRP and TH in control (*n* = 3) and organ cultured (*n* = 3) preparations (Fig. 7). Compared with control preparations, CGRP and TH immunoreactivity was markedly reduced. As shown previously (Sharrad et al., 2015), relatively weak CGRP labeling of varicosities persisted after organ culture, consistent with a population of CGRP-immunoreactive intrinsic enteric neurons (Furness et al., 2004; Smolilo et al., 2020). Intensely CGRP-immunofluorescent axons that are characteristic of the population of TRPV1-immunoreactive extrinsic afferents (Sharrad et al., 2015) were absent after organ culture. Virtually no TH immunoreactivity persisted after organ culture, consistent with ablation of sympathetic efferent axons. In addition, neither CGRP nor TH co-labeled with biotinamide labeled axons of the recorded nerve trunks after organ culture (*n* = 3). These data indicate spinal afferent and sympathetic efferent neurons whose axons lacked nerve cell bodies degenerated in organ cultured preparations while viscerofugal neurons whose axons remained attached to nerve cell bodies survived (Hibberd et al., 2012a). This is consistent with the lack of capsaicin-evoked firing in organ cultured preparations, and the origin of firing recorded from rectal nerves in organ cultured preparations being viscerofugal neurons.

### Sympathetic efferent firing during the motor complex

**Figure 8.**
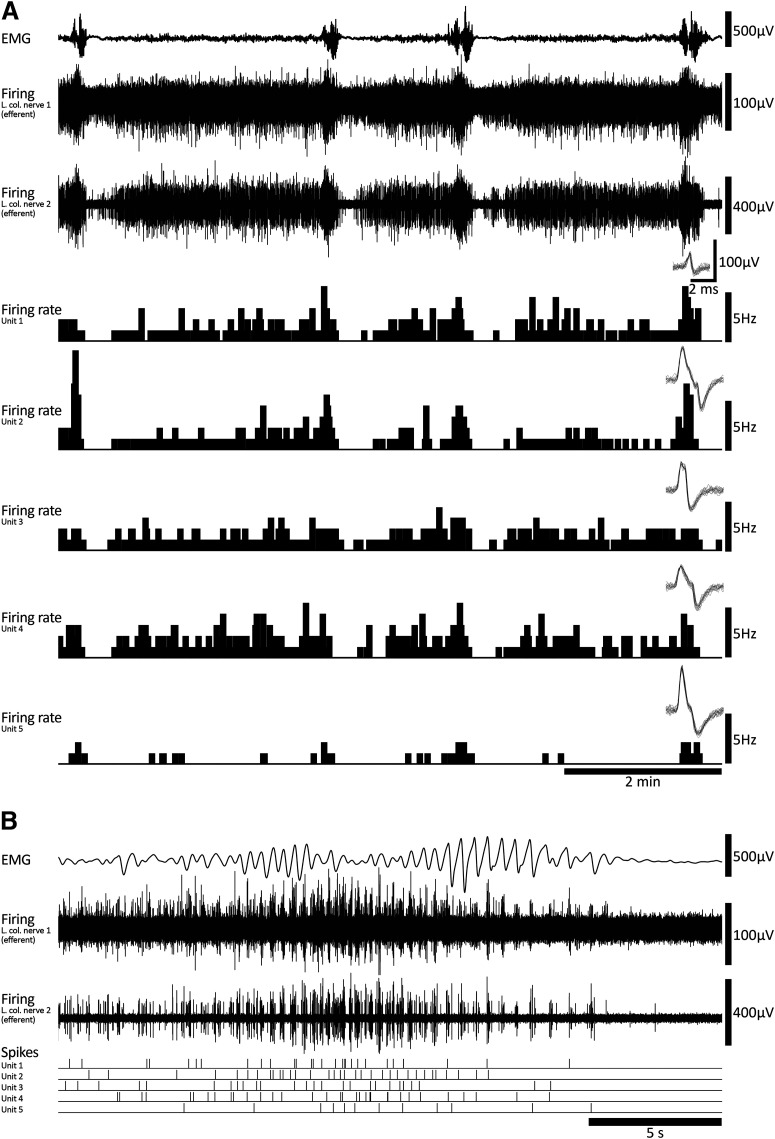
Sympathetic neuron firing and motor complexes. ***A***, Colonic EMG and lumbar colonic nerve recordings (central efferent side) showing ongoing motor complexes over ∼10 min. Sympathetic firing increased during each motor complex. ***B***, A single motor complex showing sympathetic burst firing at close to 2 Hz and single unit spikes.

**Figure 9.**
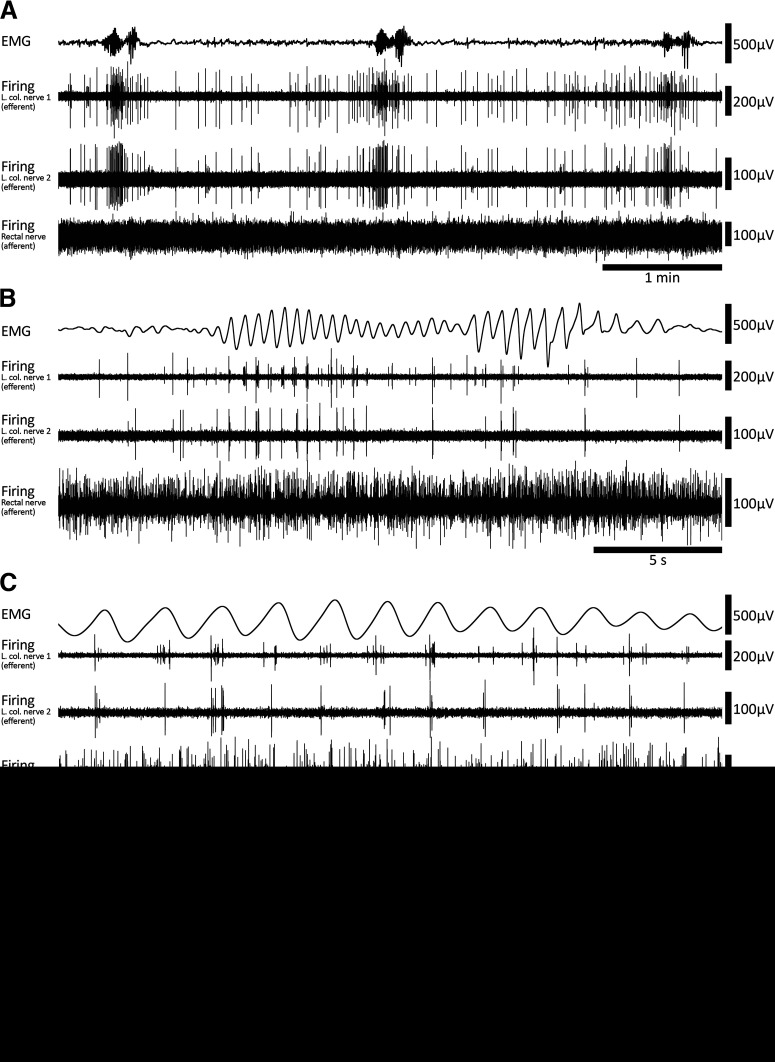
Simultaneous afferent/efferent nerve recording and the abolition of lumbar colonic nerve efferent firing by hypogastric nerve crush. ***A***, Example of three motor complexes and associated firing in two efferent recordings (lumbar colonic nerves) and a single afferent recording (rectal nerve). ***B***, The second motor complex shown in ***A*** on an expanded timescale. Note the coordination of efferent firing between the two lumbar colonic nerves. Some burst firing activity in the rectal nerve (afferent) can be seen among dense ongoing firing. ***C***, Part of the same event shown in ***B*** with an expanded timescale. At this scale both afferent (rectal nerve) and efferent (lumbar colonic nerves) burst firing can be visualized. ***D***, Abolition of efferent nerve firing by hypogastric nerve crush. Motor complexes persisted following hypogastric nerve crush, without associated bursts of sympathetic neuron firing.

Viscerofugal neurons provide fast nicotinic synaptic inputs to prevertebral sympathetic neurons (Szurszewski and King, 1989). To study whether viscerofugal inputs can independently drive postganglionic sympathetic neuron firing in the absence of preganglionic input, ex vivo preparations of whole colon were setup with intact connections to the decentralized inferior mesenteric ganglion (Fig. 1*B*). Nerve firing was recorded from the central (efferent) side of cut lumbar colonic nerve trunks, close to the mesenteric ganglion, presumed to represent activity of the postganglionic sympathetic neurons to the colon, with a simultaneous extracellular smooth muscle recording to detect CMCs (18 single units, six nerve trunks, *n* = 4; Fig. 1*B*). Ongoing spontaneous CMCs were detected in all preparations with an average frequency of 0.22 ± 0.07 cpm, featuring voltage oscillations and action potentials (2.18 ± 0.13 Hz, 23.7 ± 3.6 s in duration). A large discharge of sympathetic firing in lumbar colonic nerves occurred during each CMC and was typically followed by a modest reduction of firing after CMCs (Fig. 8). The average single unit firing rate before CMCs was 0.7 ± 0.4 Hz, 1.6 ± 0.8 Hz during the CMC, and 0.5 ± 0.3 Hz immediately after CMCs (*p* = 0.023, one-way repeated measures ANOVA, *n* = 4). Sympathetic neuron firing during the CMC was organized into sequences of synchronized bursts with a mean frequency of 2.22 ± 0.08 Hz, similar to activity shown by viscerofugal neurons. Indeed, simultaneous recording of colonic viscerofugal afferent and sympathetic efferent firing was performed in two of four preparations (Fig. 1*B*), revealing both viscerofugal burst firing in rectal nerves and sympathetic burst firing in lumbar colonic nerves (Fig. 9). Increases in sympathetic neuron firing during the CMC persisted even after smooth muscle paralysis by 3 μm nicardipine in 3/3 preparations tested. Firing was abolished by crushing the hypogastric nerves (2/2 preparations tested; Fig. 9), confirming the pathway as a source of peripheral input to the inferior mesenteric ganglion (Job and Lundberg, 1952; Crowcroft and Szurszewski, 1971).

## Discussion

This study reveals a novel ENS-derived mechanism by which sympathetic gut reflexes are activated. In this mechanism, the rhythmic ENS-generated firing pattern that underlies CMCs and drives characteristic rhythmic electrical activity in smooth muscle (Spencer et al., 2018) is also transmitted to prevertebral ganglia by viscerofugal neurons. Thus, sympathetic reflexes accompany CMC generation. Reflex activation did not require muscle contraction or dynamic changes in gut wall circumference. Importantly, the ∼2-Hz burst firing pattern underlying CMCs coordinated firing of multiple viscerofugal neurons, causing activation of sympathetic neurons with a similar 2-Hz firing pattern, even without central preganglionic input. Viscerofugal neurons predominantly supply subthreshold synaptic inputs to sympathetic neurons. Thus, the synchronizing mechanism provided by the ENS that enables assemblies of viscerofugal neurons to fire concurrently may be critical for their ability to evoke parallel firing in sympathetic neurons. The present results suggest a major revision of our conceptual understanding of sympathetic reflexes to the gut is required, which are currently considered fundamentally sensory in nature, but are here shown to be driven by highly organized output from the ENS.

Early studies revealed spinal intestino-intestinal reflex pathways (Youmans, 1944; Furness and Costa, 1974; Szurszewski and Miller, 1994). Such pathways were activated by intense intestinal distension and chemical stimuli and were regarded as a defense response to intestinal insults (Bayliss and Starling, 1899; Cannon and Murphy, 1906; King, 1924; Pearcy and Liere, 1926; Markowitz and Campbell, 1927; Douglas and Mann, 1941; Chang and Hsu, 1942). The later discovery of enteric viscerofugal inputs to prevertebral sympathetic ganglia was shown functionally (Garry, 1933; Lawson, 1934; Lawson and Holt, 1937; Kuntz, 1940; Kuntz and Van Buskirk, 1941; Lium and Portsmouth, 1941; Kuntz and Saccomanno, 1944; Crowcroft et al., 1971; Weems and Szurszewski, 1977; Kreulen and Szurszewski, 1979) and anatomically (Ross, 1958; Schofield, 1960; Ungvary and Leranth, 1970; Dalsgaard and Elfvin, 1982; Feher, 1982; Kuramoto and Furness, 1989; Messenger and Furness, 1991, 1992). These findings suggested a parallel mechanism of sympathetic intestinal inhibition that bypassed the CNS (Furness and Costa, 1974).

Mechanical and electrophysiological investigations of sympathetic prevertebral reflexes in colon report sympathetic neurons are activated during distension (Crowcroft et al., 1971; Szurszewski and Weems, 1976; Weems and Szurszewski, 1977; Kreulen and Szurszewski, 1979; Kreulen and Peters, 1986; Peters and Kreulen, 1986; Anthony and Kreulen, 1990; Parkman et al., 1993; Bywater, 1994; Stebbing and Bornstein, 1994; Miller and Szurszewski, 1997, 2002, 2003; Ermilov et al., 2004). Where nicotinic or synaptic blockade in the gut has been applied, evidence suggests viscerofugal neurons are directly mechanosensitive (Parkman et al., 1993; Bywater, 1994; Stebbing and Bornstein, 1994), and firing correlates more closely with gut length (volume) than tension (Hibberd et al., 2012b; Palmer et al., 2016). However, where ENS cholinergic transmission is permitted, the higher-order role of viscerofugal neurons, and their relationship to gut mechanical activity is unclear. Most viscerofugal neuron input to sympathetic neurons is synaptically driven by other enteric neurons (Crowcroft et al., 1971; Miller and Szurszewski, 1997) and is therefore important for sympathetic reflex activation. Indeed, direct intracellular recordings from guinea pig colonic viscerofugal neurons show they receive nicotinic inputs from multiple myenteric neural pathways (Sharkey et al., 1998; Hibberd et al., 2014), and immunohistochemical analyses suggest myenteric descending interneurons contribute most cholinergic terminals to viscerofugal nerve cell bodies (Lomax et al., 2000). Thus, viscerofugal neurons are situated to receive outputs of enteric neural pathways. Nevertheless, the idea that firing transmitted from viscerofugal neurons encodes sensory information about the gut wall (particularly gut volume) during sympathetic reflexes remains dominant.

Here, periodic firing transmitted by viscerofugal neurons to sympathetic neurons during CMCs persisted with constant gut wall length and smooth muscle paralysis, indicating the activity did not encode mechanosensory information about the gut wall. Rather, we hypothesize viscerofugal enteric neuron firing was closely related to circuits that generate CMCs. Cholinergic-nicotinic neurotransmission is the principle form of fast excitatory neurotransmission in the ENS (Furukawa et al., 1986; Nurgali et al., 2004) and thus most likely to drive viscerofugal neuron firing. However, nicotinic transmission is also required for CMCs in mouse colon (Bywater et al., 1989; Lyster et al., 1995; Spencer et al., 1998a,b; Bush et al., 2000). We suggest this explains the concomitant abolition of both viscerofugal neuron burst firing and CMCs in the present study. However, the results do not rule out a possibility that burst firing is driven by a non-nicotinic neural mechanism during CMCs (Bian et al., 2003; Nurgali et al., 2004).

Most myenteric neurons fire during CMCs at ∼2 Hz, driving smooth muscle firing at the same frequency (Spencer et al., 2018). The observation that viscerofugal neurons also fire at this frequency during CMCs suggest they receive input from circuitry that generate CMCs. This is compatible with evidence viscerofugal neurons receive inputs from enteric interneurons, as is the observation that multiple viscerofugal neurons fired together in brief bursts, which may be explained by a coordinating mechanism provided by common interneuronal connections. Also, individual viscerofugal neurons had a high probability of not contributing action potentials to individual firing bursts during CMCs (Figs. 4, 5*B*). Thus, while assemblies of multiple viscerofugal neurons clearly showed burst firing output at 2 Hz during CMCs, individual neurons made partial contributions to the overall behavior. The output of viscerofugal neuron assemblies thus differed from their individual contributions, highlighting the role of synchronization among viscerofugal neurons in producing a coherent burst firing pattern.

Some studies report firing behavior that also challenge the idea viscerofugal neurons simply relay mechanosensory information about volume. In guinea pig colon, viscerofugal neurons showed large bursts of firing that preceded spontaneous and stretch-evoked contractions after organ-culture (Hibberd et al., 2012a). In mouse colon, large changes in synaptic input frequency to sympathetic neurons were observed during isovolumetric colonic contractions (Miller and Szurszewski, 2002). Isovolumetric distension is closely analogous to the maintained isometric conditions used in the present study. We hypothesize that in the elegant study of Miller and Szurszewski (2002), bursts of synaptic input to sympathetic neurons during intracolonic filling were not dependent on filling per se, but on the ENS activity associated with neurogenic contractions elicited by colonic filling. A major difference here was use of pharmacological smooth muscle paralysis, enabling isolation of periodic activation of viscerofugal and sympathetic neurons from dynamic mechanical activities of the gut wall. Thus, sympathetic reflexes evoked during gut motor activity do not simply reflect mechanosensory encoding by viscerofugal neurons but rather, transmit the functional state of ENS motor circuits. Thus, sympathetic reflexes through viscerofugal neurons may represent negative feedback loops for long-range self-regulation of ENS excitability.

The CMC in mouse is a self-organized motor pattern that emerges from the ENS under maintained distension. CMCs occur rarely without distension, and frequency is graded with increasing amounts of distension (Barnes et al., 2014; Hibberd et al., 2017). Thus, sustained increases in gut volume represent an effective initiator of CMCs and this may explain much of the association with synaptically-driven viscerofugal neuron firing. Additionally, while CMCs may occur without propulsive movements, as in this study, the ENS-sympathetic peripheral loop may also be activated during propulsive contractions, since they feature similar coordinated 2-Hz firing in smooth muscle (Spencer et al., 2018).

Interestingly, sympathetic neurons were activated during colonic CMCs in decentralized inferior mesenteric ganglia. This suggests that intestino-intestinal reflexes may occur entirely peripherally and does not require ongoing excitation by spinal preganglionic neurons. As noted, viscerofugal neurons provide numerous “weak” (i.e., subthreshold) nicotinic synaptic inputs to sympathetic prevertebral neurons (McLachlan and Meckler, 1989; Miller and Szurszewski, 1997; McLachlan, 2003). To elicit firing, weak synapses require integration of multiple inputs via temporal and spatial summation. The temporally synchronized discharge of viscerofugal neurons identified in the present study may therefore be significant for determining activation of sympathetic neurons via the well-established mechanisms of temporal summation. Indeed, the appearance of large excitatory postsynaptic currents in postganglionic neurons of mouse superior mesenteric ganglion during colonic distension raised the question whether viscerofugal inputs could be synchronized (Skok et al., 1998). The present study argues in favor of this explanation.

Additional sources of input to prevertebral ganglia are axon collaterals of spinal sensory neurons, which synapse en passant (Matthews and Cuello, 1982, 1984; Matthews et al., 1987). During organ culture, extrinsic sensory and sympathetic axons degenerate because they are cut off from their cell bodies. Thus, the conclusion that enteric viscerofugal neurons mediated burst firing behavior recorded from rectal nerves during motor complexes in organ cultured preparations cannot be escaped. However, it may be suggested, at least in control preparations with intact spinal afferent neurons, that spinal afferent axons branching in prevertebral ganglia potentially activated postganglionic sympathetic neurons in a mechanism similar to the “Sokownin reflex” (Sokownin, 1877; Job and Lundberg, 1952; Bulygin, 1983). Spinal afferent collaterals contain peptide transmitters that can be released during gut distension causing slow depolarization of sympathetic neurons (Dun and Karczmar, 1979; Dun and Jiang, 1982; Peters and Kreulen, 1986; Webber and Heym, 1988; Dun and Mo, 1989; Ma and Szurszewski, 1996; Jobling and Gibbins, 1999; Kaestner et al., 2019). Slow depolarizations range seconds to minutes in duration, making this mode unlikely to account for the high-fidelity transmission of the ∼2-Hz burst firing pattern from the gut to sympathetic neurons during CMCs. However, it is possible that activation of spinal afferent collateral branches in sympathetic ganglia, can further enhance ENS-mediated excitation of sympathetic neurons, in addition to participation in long spinal intestino-intestinal inhibitory reflexes (Youmans, 1944; Furness and Costa, 1974; Szurszewski and Miller, 1994).

In guinea pig, viscerofugal neurons selectively innervate sympathetic motor and secretomotor neurons but not vasoconstrictor neurons (Costa and Furness, 1984; Macrae et al., 1986; Meckler and McLachlan, 1988; Gibbins et al., 2003). The specificity of viscerofugal targets remain to be confirmed in mouse and there is currently no useful chemical coding scheme identified among sympathetic neurons in mouse to differentiate functional subtypes (Kaestner et al., 2019). However, we speculate the viscerofugal-sympathetic loop ensures CMCs cause inhibition of motility and secretion further oral along the gut (Messenger and Furness, 1993; Furness et al., 2000; Furness, 2003). CMCs often propagate along most of the length of colonic preparations isolated from prevertebral ganglia and may do so at high apparent velocities in either direction (Fida et al., 1997; Costa et al., 2017). One implication of sympathetic reflex activation during CMCs may be a limitation of ENS excitability to shorter gut lengths, leading to shorter CMC events. Retrograde CMCs may be particularly limited by orally-directed sympathetic reflexes. These possibilities remain to be investigated experimentally.

In summary, we found that activation of hardwired ENS-sympathetic reflexes accompanied physiological motor behavior in mouse colon. Assemblies of viscerofugal neurons fired concurrently in bursts at a frequency of ∼2 Hz, leading to parallel activation of sympathetic neurons in lumbar colonic nerves at a similar frequency. Since the activity transmitted from the ENS to sympathetic ganglia is highly coordinated, it is likely to represent a powerful mechanism, involving interneuronal processing, by which sympathetic ganglia read out the functional state of ENS motor circuits.

## Synthesis

Reviewing Editor: Quentin Pittman, University of Calgary Decisions are customarily a result of the Reviewing Editor and the peer reviewers coming together and discussing their recommendations until a consensus is reached. When revisions are invited, a fact-based synthesis statement explaining their decision and outlining what is needed to prepare a revision will be listed below. The following reviewer(s) agreed to reveal their identity: Kirsteen Browning, James Galligan. Note: If this manuscript was transferred from JNeurosci and a decision was made to accept the manuscript without peer review, a brief statement to this effect will instead be what is listed below.

The manuscript by Hibberd et al., used extracellular recording techniques to re-examine the activity of viscerofugal neurons during ongoing colonic motility patterns. The authors note that ongoing colonic motor contractile activity induced synchronized activity in viscerofugal fibers, an activity pattern that was confirmed by recordings in organtypic culture. Further analyses noted that the nerve firing patterns arises from assemblies or groups of viscerofugal neurons implied synchronization of enteric neuronal activity, the pattern of which was replicated in sympathetic neurons. The reviewers note that this is a complex study with multiple moving parts and difficult recording techniques

The function and activity of viscerofugal neurons has received little attention, despite being known for several decades that their activity is important in the co-ordination of enteric functions and communication with extrinsic neural systems. The preparation used in these studies mimics previous work where the active colon remained connected to the inferior mesenteric ganglion (IMG) during CMCs. Using this technique spontaneous or electrically-evoked cholinergic fEPSPs could be recorded from sympathetic neurons in the attached IMG (Anthony and Kreulen, 1990; Miller and Szurszewski, 1997). Other investigators have also used fluorescent tracers to track the nerve endings of the sympathetic nerves originating in the IMG or tracking nerve endings of the intestinofugal neurons originating in the colonic myenteric plexus. Many others have studied CMCs and the reciprocal connections between the myenteric plexus and the IMG in guinea pigs with similar results.

Hexamethonium abolished CMC and burst firing (suggesting involvement of nicotinic receptor and/or muscle contractility) whereas nicardipine abolished CMC but not burst firing (suggesting burst firing was not dependent upon muscle activity). The response to capsaicin in flat sheet preparations may be harder to interpret given TRPV1 receptors are expressed on gastrointestinal smooth muscle as well as enteric neurons, but when coupled with the lack of effect of capsaicin on burst firing responses in organotypic culture preparations (where spinal afferents are reasonably assumed to have degenerated, although additional demonstrated confirmation in this manuscript would be helpful) implies that the burst firing is independent from CMC. Why do the authors conclude that the burst firing activity is “either dependent upon nicotinic input, CMCs, or both” when the results present suggest the sole involvement of neuronal, not muscle, activity?

The authors conclude that viscerofugal inputs drive postganglionic sympathetic neuron activity because (i) sympathetic activity was synchronized with CMC activity, (ii) sympathetic activity was similar in frequency to viscerofugal fiber activity, and (iii) firing was abolished by crushing hypogastric nerves. This does not necessarily eliminate the possible contribution of axon reflexes via the peripheral fibers of sympathetic neurons. Demonstrating the dependence on specifically synaptic viscerofugal activity (either localized application of nicotinic antagonists or synaptic blockers, and or selective interruption of descending myenteric interneuron pathways thought to contribute significantly to viscerofugal neuron activity) would eliminate the involvement of a purely axon reflex and add credence to the authors conclusions of viscerofugal neuron involvement. If under the current COVID conditions you are able to address this experimentally, we feel your paper would be much stronger; if not please address the potential involvement of axon reflexes in the discussion.

## Author Response

Dear Professor Pittman,

We thank you and the reviewers for the positive comments and suggestions regarding our manuscript. Several questions were contained in the synthesis statement that we have sought to address below and which have prompted revisions that we feel have improved the manuscript. We bring to your attention correspondence from Christophe Bernard proposing the use of estimation statistics in our manuscript (Ho et al., 2019). This approach has been implemented where possible, resulting in the additional figures 5C-5G, and adjustments to values in the text on pages 12 and 13 in the revised manuscript. All changes are shown in bold text, as required. We hope these revisions meet your expectations and again thank you for your time.

Nick Spencer

Hexamethonium abolished CMCs and burst firing (suggesting involvement of nicotinic receptor and/or muscle contractility) whereas nicardipine abolished CMC but not burst firing (suggesting burst firing was not dependent upon muscle activity). The response to capsaicin in flat sheet preparations may be harder to interpret given TRPV1 receptors are expressed on gastrointestinal smooth muscle as well as enteric neurons, but when coupled with the lack of effect of capsaicin on burst firing responses in organotypic culture preparations (where spinal afferents are reasonably assumed to have degenerated, although additional demonstrated confirmation in this manuscript would be helpful) implies that the burst firing is independent from CMC. Why do the authors conclude that the burst firing activity is “either dependent upon nicotinic input, CMCs, or both” when the results present suggest the sole involvement of neuronal, not muscle, activity?

We agree the results suggest a neural dependence and organization of the burst firing behaviour among viscerofugal and sympathetic neurons during the CMC. We failed to make it clear that the neural activity underlying CMCs persists when the smooth muscle contractions are blocked by nicardipine; this has now been clarified on page 12. As noted in the question, the lack of effect of nicardipine enables us to rule out that viscerofugal/sympathetic burst firing depends on smooth muscle contractions. However, the interpretation of hexamethonium effects are more complicated. This drug blocks nicotinic input to viscerofugal neurons (the putative major source of input to viscerofugal neurons) and also reliably abolishes the CMCs (e.g. Bywater et al., 1989, Lyster et al., 1995, Spencer et al., 1998a, Spencer et al., 1998b, Bush et al., 2000). This leaves open the possibility that inhibition of viscerofugal neuron firing by hexamethonium was indirect. We cannot rule out that CMC circuits might activate viscerofugal neurons via non-nicotinic synaptic inputs and that effects of hexamethonium on viscerofugal neurons were due to blockade of CMCs. However, we believe that the most likely case is that both CMCs and viscerofugal burst firing behaviour require on nicotinic synaptic transmission. This has been clarified in the revised manuscript on pages 18 and 19.

Finally, the referee suggested that additional evidence supporting spinal afferent ablation in organ-cultured preparations would be desirable. We have conducted additional experiments to provide immunohistochemical assessment of common markers of extrinsic neurons, CGRP (for sensory axons) and TH (for sympathetic axons) comparing cultured and control preparations. Similar to previous published results in the guinea pig (Hibberd et al., 2012), both CGRP and TH were largely depleted after organ culture, consistent with the degeneration of extrinsic afferent and efferent nerve fibres. Intensely labelled CGRP axons were almost absent from organ-cultured preparations. As we have described previously (Sharrad et al., 2015), varicose axons that are relatively weakly immunofluorescent for CGRP persisted after culture and probably represent a population of intrinsic enteric neurons that express CGRP (Furness et al., 2004). We have added these results on page 15, representative images in Figure 7, and methodology on page 9 of the revised manuscript.

The authors conclude that viscerofugal inputs drive postganglionic sympathetic neuron activity because (i) sympathetic activity was synchronized with CMC activity, (ii) sympathetic activity was similar in frequency to viscerofugal fiber activity, and (iii) firing was abolished by crushing hypogastric nerves. This does not necessarily eliminate the possible contribution of axon reflexes via the peripheral fibers of sympathetic neurons. Demonstrating the dependence on specifically synaptic viscerofugal activity (either localized application of nicotinic antagonists or synaptic blockers, and or selective interruption of descending myenteric interneuron pathways thought to contribute significantly to viscerofugal neuron activity) would eliminate the involvement of a purely axon reflex and add credence to the authors conclusions of viscerofugal neuron involvement.

This is an interesting suggestion. During organ culture, extrinsic sensory and sympathetic axons degenerate because they are cut off from their cell bodies. The detection of burst firing in organ cultured tissue thus neccessarily implicates enteric viscerofugal neurons, and suggests the idea that collaterals of sympathetic neurons contributed to the firing behaviour observed in organ cultured preparations is unlikely. Furthermore, there is no evidence that sympathetic neurons can be physiologically activated by any mechanism other than synaptic input onto their nerve cell bodies or dendrites. Despite this, our studies cannot entirely rule out axon reflexes via the peripheral fibers of sympathetic neurons, contributing to the activity detected in control preparations. However, previous intracellular recordings of sympathetic nerve cell bodies in superior mesenteric ganglion attached to the colon showed that nicotinic blockade applied selectively to the ganglion abolished ongoing and distension-evoked action potentials (Miller and Szurszewski, 1997). This argues axon reflexes from the gut in sympathetic prevertebral neurons are either rare, or they are undetectable from the nerve cell body. It may be suggested, at least in control preparations where spinal afferent axons remained intact, that spinal afferent axons branching in prevertebral ganglia potentially activated postganglionic sympathetic neurons in a mechanism similar to the “Sokownin reflex” (Job and Lundberg, 1952). However, their mode of transmission is mediated by slow potentials (Dun and Jiang, 1982, Dun and Kiraly, 1983, Peters and Kreulen, 1986) and thus unlikely to the evoke 1:1 the ∼2Hz burst firing pattern in sympathetic neurons. We have added this to the discussion on page 21 of the revised manuscript.

-

Bush TG, Spencer NJ, Watters N, Sanders KM, Smith TK (2000) Spontaneous migrating motor complexes occur in both the terminal ileum and colon of the C57BL/6 mouse in vitro. Autonomic Neuroscience 84:162-168.

Bywater RA, Small RC, Taylor GS (1989) Neurogenic slow depolarizations and rapid oscillations in the membrane potential of circular muscle of mouse colon. The Journal of Physiology 413:505-519.

Dun N, Jiang Z (1982) Non-cholinergic excitatory transmission in inferior mesenteric ganglia of the guinea-pig: possible mediation by substance P. The Journal of Physiology 325.

Dun N, Kiraly M (1983) Capsaicin causes release of a substance P-like peptide in guinea-pig inferior mesenteric ganglia. The Journal of Physiology 340:107.

Furness JB, Robbins HL, Xiao J, Stebbing MJ, Nurgali K (2004) Projections and chemistry of Dogiel type II neurons in the mouse colon. Cell and Tissue Research 317:1-12.

Hibberd TJ, Zagorodnyuk VP, Spencer NJ, Brookes SJH (2012) Viscerofugal neurons recorded from guinea-pig colonic nerves after organ culture. Neurogastroenterology and Motility 24:1041-e1548.

Ho J, Tumkaya T, Aryal S, Choi H, Claridge-Chang A (2019) Moving beyond P values: data analysis with estimation graphics. Nat Methods 16:565-566.

Job C, Lundberg A (1952) Reflex excitation of cells in the inferior mesenteric ganglion on stimulation of the hypogastric nerve. Acta Physiologica Scandinavica 26:366-382.

Lyster DJK, Bywater RAR, Taylor GS (1995) Neurogenic control of myoelectric complexes in the mouse isolated colon. Gastroenterology 108:1371-1378.

Miller SM, Szurszewski JH (1997) Colonic mechanosensory afferent input to neurons in the mouse superior mesenteric ganglion. American Journal of Physiology-Gastrointestinal and Liver Physiology 272:G357-366.

Peters S, Kreulen DL (1986) Fast and slow synaptic potentials produced in a mammalian sympathetic ganglion by colon distension. Proceedings of the National Academy of Sciences of the United States of America 83:1941-1944.

Sharrad DF, Hibberd TJ, Kyloh MA, Brookes SJH, Spencer NJ (2015) Quantitative immunohistochemical co-localization of TRPV1 and CGRP in varicose axons of the murine oesophagus, stomach and colorectum. Neuroscience Letters 599:164-171.

Spencer NJ, Bywater RA, Klemm MF (1998a) Effects of sympathetic nerve stimulation on membrane potential in the circular muscle layer of mouse distal colon. Neurogastroenterol Motil 10:543-552.

Spencer NJ, Bywater RAR, Taylor GS (1998b) Disinhibition during myoelectric complexes in the mouse colon. Journal of the Autonomic Nervous System 71:37-47.
